# Quantification of Differential Response of Tumour and Normal Cells to Microbeam Radiation in the Absence of FLASH Effects

**DOI:** 10.3390/cancers13133238

**Published:** 2021-06-29

**Authors:** Harriet Steel, Sarah C. Brüningk, Carol Box, Uwe Oelfke, Stefan H. Bartzsch

**Affiliations:** 1 Division of Radiotherapy and Imaging, The Institute of Cancer Research, 123 Old Brompton Road, London SW7 3RP, UK; carol.box@icr.ac.uk (C.B.); uwe.oelfke@icr.ac.uk (U.O.); 2 Machine Learning & Computational Biology, Department of Biosystems Science and Engineering, ETH Zurich, 4058 Basel, Switzerland; sarah.brueningk@bsse.ethz.ch; 3 Swiss Institute for Bioinformatics (SIB), 1015 Lausanne, Switzerland; 4 Department of Radiation Oncology, School of Medicine, Klinikum Rechts der Isar, Technical University of Munich, Ismaninger Straße 22, 81675 Munich, Germany; stefan.bartzsch@tum.de; 5 Helmholtz Centre Munich, Institute for Radiation Medicine, Ingolstädter Landstraße 1, 85764 Munich, Germany

**Keywords:** microbeam, in vitro, compact source, clonogenic survival, integral dose, LQ model, spatial fractionation

## Abstract

**Simple Summary:**

This study compares the capabilities of normal and tumour cells to divide after receiving spatially fractionated radiotherapy. For this treatment, a minority of cells (here ∼20%) receive very large, peak doses, whereas the remaining cells are spared and receive only a small valley dose. We show that tumour and normal cells respond differently to this treatment, and that spatially fractionated radiotherapy may have a greater effect on tumour than normal cells. Our work was conducted using laboratory equipment, rather than specialized synchrotron facilities implying that the observed response is present at conventional dose rates and hence purely an effect of the spatial fractionation of the treatment.

**Abstract:**

Microbeam radiotherapy (MRT) is a preclinical method of delivering spatially-fractionated radiotherapy aiming to improve the therapeutic window between normal tissue complication and tumour control. Previously, MRT was limited to ultra-high dose rate synchrotron facilities. The aim of this study was to investigate in vitro effects of MRT on tumour and normal cells at conventional dose rates produced by a bench-top X-ray source. Two normal and two tumour cell lines were exposed to homogeneous broad beam (BB) radiation, MRT, or were separately irradiated with peak or valley doses before being mixed. Clonogenic survival was assessed and compared to BB-estimated surviving fractions calculated by the linear-quadratic (LQ)-model. All cell lines showed similar BB sensitivity. BB LQ-model predictions exceeded the survival of cell lines following MRT or mixed beam irradiation. This effect was stronger in tumour compared to normal cell lines. Dose mixing experiments could reproduce MRT survival. We observed a differential response of tumour and normal cells to spatially fractionated irradiations in vitro, indicating increased tumour cell sensitivity. Importantly, this was observed at dose rates precluding the presence of FLASH effects. The LQ-model did not predict cell survival when the cell population received split irradiation doses, indicating that factors other than local dose influenced survival after irradiation.

## 1. Introduction

Any cancer treatment aims to eradicate the tumour target, whilst inflicting minimal toxicity in healthy tissues. In radiation therapy (RT), this aim is conventionally achieved by geometrically confining the high dose field to the tumour, e.g., by intensity-modulated RT, and thereby limiting side effects to organs at risk (OAR). However, exposure of OARs located in close proximity to the tumour, or along the beam path, is inevitable and limits the dose escalation to the tumour with potential implications on outcome. Spatially fractionated RT, such as microbeam radiation therapy (MRT) [1], has previously been suggested as an alternative strategy to maximise the therapeutic window between tumour control and normal tissue complication probability. MRT uses arrays of planar, high-dose beams of tens of µm width, which are separated by a few hundred micrometres. This spatial fractionation results in small regions of tissue receiving large (generally 300–800 Gy) peak doses being ablated, whereas spared areas receive a several-fold lower (valley) dose. In order to maintain the collimated dose pattern, keV photon beams are employed for MRT delivery typically produced at large third-generation synchrotrons to prevent motion blurring of the spatial dose pattern through high photon flux delivery.

Preclinical in vivo data have demonstrated a remarkable normal tissue sparing following MRT, despite peak doses in the range of hundreds of grays [2,3,4,5,6,7,8]. It has also been shown that MRT is effective for the treatment of tumours in preclinical models of brain cancer [9,10,11,12,13,14] and melanoma [15]. Together, these studies suggest that MRT has a differential effect on normal and tumour tissues, indicating its high therapeutic potential for cancer treatment. Currently, the origin of the differential effect of MRT on tumour and normal tissue (referred to as "the microbeam effect") is a matter of scientific debate. Hypotheses proposed include a role for vascular maturity [5,12,16,17,18,19], the immune system [20,21,22,23,24] and bystander effects [25,26]. More recently, however, there is growing evidence for normal tissue sparing through the use of ultra-high dose rates delivered at synchrotron facilities (FLASH) [27,28]. Hence, it remains to be seen how much of the normal tissue sparing, previously attributed to MRT is indeed a result of spatial dose fractionation as opposed to FLASH effects.

Moreover, there is little data to support the existence of the microbeam effect in vitro [26,29,30], i.e., in the absence of immune system- or vascular-mediated effects. A differential response to MRT in normal and tumour cells in vitro would indicate a role for additional components, such as bystander signalling. Previous work on MRT evaluation in vitro either lacked a detailed comparison of normal and tumour cells [29,31], or did not evaluate MRT in relation to conventional BB irradiation. A possible reason may be difficulties in comparing the highly heterogeneous dose profiles of MRT to BB for this purpose. In light of the linear-quadratic relation of cell survival with radiation dose, it is clear that neither mean, peak nor valley dose alone are sufficient for a comparison with BB treatments.

In this study, we evaluate the response of two tumour and two non-tumour human cell lines in response to BB and MRT to investigate and quantify differential effects of these treatments delivered with a conventional X-ray tube and optional MRT collimation [32]. As such, our system precludes the presence of FLASH effects implying that any differential would exclusively be attributed to spatial fractionation. Moreover, by means of calculating cell survival using BB linear quadratic model parameters and the MRT dose distribution, we are able to draw a direct comparison between BB and MRT treatment efficacies.

## 2. Materials and Methods

### 2.1. Cell Culture

Human umbilical vein endothelial cells (HUVEC) from pooled donors were purchased from Lonza (Slough, UK) and MRC-5 normal lung fibroblasts, from Sigma Aldrich Ltd. (Merck KGaA, Darmstadt, Germany). For the purposes of this manuscript, we will refer to these as “normal” cells. The human non-small cell lung cancer lines A549 and NCI-H23 were obtained from The American Type Culture Collection (ATCC, Gaithersburg, USA). Tumour cell lines were cultured in Dulbecco’s minimal essential medium F12 (Gibco Life Technologies Ltd., Paisley, UK), and MRC-5 cells were cultured in minimal essential medium (MEM; Gibco Life Technologies Ltd), both supplemented with 10% foetal bovine serum (PAN Biotech GmbH, Aidenbach, Germany). HUVECs were cultured in endothelial cell growth medium-2 (Lonza), including all supplements supplied by the manufacturer. Cells were maintained in a humidified incubator at 37 °C and 5% CO_2_. Screening for mycoplasma contamination was performed by polymerase chain reaction (Surrey Diagnostics, Cranleigh, UK) and cell lines were authenticated in-house by short tandem repeat analysis using a Gene Print 10.0 kit (Promega, Madison, USA) and a 3730xl DNA analyser (Applied Biosystems, Warrington, UK).

### 2.2. Clonogenic Assay

To ensure that all clonogenic assays were carried out on exponentially growing cells, cells were seeded at approximately 16 h prior to RT, yielding 80% confluence at the time of irradiation. Following irradiation, cells were immediately harvested by trypsinisation, counted and plated at appropriate numbers in triplicate in 6-well plates. Cells were then incubated under the specified culture conditions and allowed to form colonies for 7–14 days, depending on the cell line. Colonies were fixed in ice-cold methanol at −20 °C for 20 min, left to dry, and stained in 0.5% crystal violet solution (Sigma Aldrich Ltd.). Colonies containing 50 or more cells were counted. Plating efficiency of the cells for each condition was calculated as the ratio of colonies counted per cell number seeded. The clonogenic survival was calculated as the ratio between plating efficiencies of treated and untreated cells. For each experiment, three independent repeats were performed, and mean values and standard deviations are reported. The cell survival data obtained are listed in Table A1, Table A2 and Table A3 in Appendix A.

### 2.3. Irradiation Procedure

For all experiments, X-rays were generated from an X-ray tube (HPZ-160-11, Varian Medical Systems, Crawley, UK) mounted in an X-ray cabinet (Xstrahl, Camberley, UK). An acceleration voltage of 160 kV, and a tube current of 11.3 mA for BB or 5.6 mA for MRT generation was used. The use of different amperage depending on the irradiation procedure was due to the use of an optimal focal spot size/tube filament yielding a more homogeneous dose distribution for BB at a high dose rate (0.031 ± 0.002 Gy/s), and a well-collimated beam with a high dose rate for MRT. The beam was hardened by 1 mm aluminium filtration resulting in a dose rate of 0.031±0.002 Gy/s at the sample position for BB exposures. MRT was generated as previously described [32]. In short, a bespoke collimator was mounted 70 mm from the source of the beam. The collimator consisted of 50 µm wide beam-slits spaced 400 µm apart.

**Figure 1:**
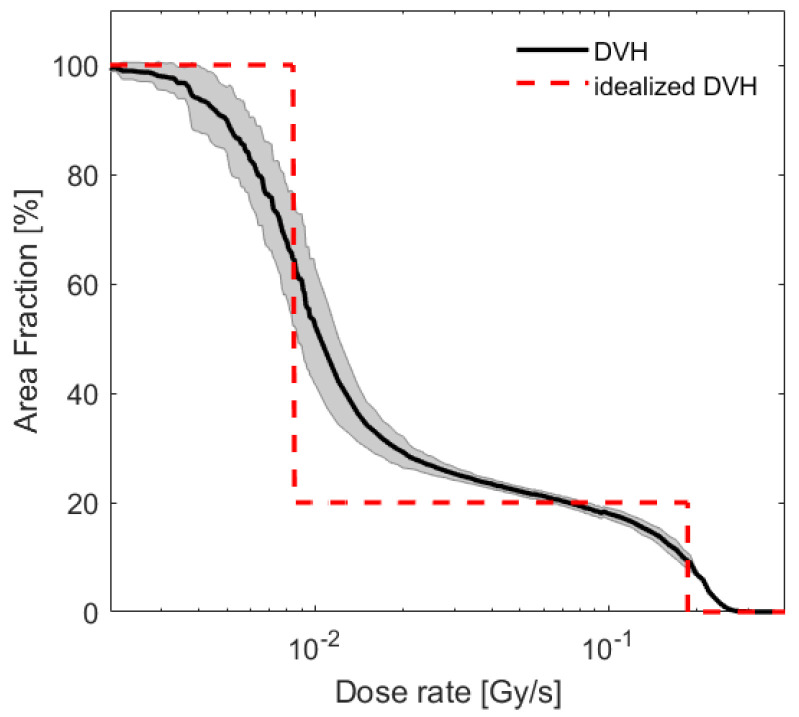
Dose rate volume histogram obtained as an average over 17 film measurements at different exposure duration (solid line). Shaded background corresponds to the standard deviation of these measurements per DVH bin. The dose distribution was approximated by an idealised, two-step DVH (dashed line) corresponding to the same integral dose rate of 0.044 Gy/s, a PVDR of 22 and a fraction of 80/20 % of cells receiving the valley/peak dose.

The MRT field was characterised following previously published procedure [33] using EBT-XD films (Gafchromic, Bridgewater, US; dynamic dose range of 0.1–60 Gy, spatial resolution of <25µm). For absolute dose measurements, calibration films were exposed to 0–100 Gy under BB conditions and correlated with ionisation chamber (Semiflex, PTW, Germany) measurements. Films were also exposed to MRT in-cell treatment geometry, i.e., accounting for equal depths of scatter material, here poly(methyl methacrylate), and air gaps. All exposed films were scanned 48 h after irradiation at 4 µm resolution with an optical microscope (Axio Scan, Zeiss, Oberkochen, Germany). Images were corrected for illumination and stitching artefacts using ZEN software (Zeiss, 2011). The dose distribution was measured in three independent experiments for MRT exposure duration between 3.5 and 12 min per film, to cover both peaks and valleys within the dynamic range of the films. Here, we visualise the obtained dose distribution in terms of a cumulative dose rate volume histogram (abbreviated as DVH, see Figure 1), which indicates the area fraction receiving at least the indicated dose rate. For a perfect MRT exposure exclusively at two dose levels (peak and valley dose) this representation would yield a step function with steps’ location on the x-axis indicating the valley (first step) and peak dose rates (second step), respectively. The step location on the y-axis indicates the area fraction receiving at least the relevant dose. The specifications of our system resulted in a heel effect across the exposed area leading to a spread of the individual peak and valley doses and deviation of the DVH from a perfect two-step distribution. For each film, a DVH was calculated and a mean DVH was generated from three independent repeat measurements, as shown in Figure 1). For each DVH bin (corresponding to a distinct dose rate), only pixels within the dynamic range of the film were included. All following data are reported as a function of this full spectrum of doses with error bars representing the standard deviation over repeat measurements.

For comparison with BB irradiation at two distinct dose levels, the experimental DVH was approximated by a step function resulting in the same integral dose as the MRT dose distribution (see Figure 1, dashed line). The coefficient of determination measured on a log scale between idealised and experimentally measured DVH was $R^{2}=0.85$, which corresponded to a peak-to-valley dose ratio (PVDR) of 22, and a spatial fraction of 80%/20% of cells receiving the valley/peak dose. This distribution was delivered for dose mixing experiments.

### 2.4. Dose Mixing

Cells were irradiated separately under BB conditions with either a peak or a valley dose, accounting for a constant PVDR of 22. Due to longer exposure duration for MRT experiments, flasks were left at room temperature after irradiation for equivalent amounts of time prior to trypsinisation. Following dissociation and counting, the cells were mixed such that 20% received the peak dose and 80% the valley dose, mimicking the results obtained from the dose rate volume histogram in Figure 1. Cell suspensions were plated for clonogenic survival, as described above.

### 2.5. Cell Survival Analysis

The dependence of clonogenic cell survival *S* on a single fraction of radiation dose *d* is conventionally described by the linear quadratic (LQ) model [34].

(1) $$S\left(d\right)=e^{-Y}=e^{-(\alpha d+\beta d^{2})}$$

Here, the biological effect, *Y* (the surviving fraction equals the exponential function *e* of −Y), characterises cell survival as a second-order polynomial of the dose *d* and the cell line and radiation quality dependent parameters α and β. For MRT a fraction $\nu (d_{i})$ of the cell culture is exposed to dose $d_{i}$, within the spectrum $\left\{d_{i}\right\}$ of *N* different doses $d_{i}\left(i=1\ldots N\right)$. Assuming that cells are homogeneously plated, this is equal to the area fraction exposed to $d_{i}$. The LQ-model predicted survival fraction $S_{pred}$ is then calculated as follows:

(2) $$S_{pred}\left(\nu \left(\left\{d_{i}\right\}\right)\right)=\sum_{i}^{N}\nu \left(d_{i}\right)\cdot S\left(d_{i}\right)$$

The clonogenic survival in response to BB irradiation was fitted to the LQ-model in MATLAB (version 2017a) using a nonlinear least square approach resulting in α and β values for each cell line. For MRT and mixing experiments, cell survival was predicted according to Equation (2) using the α and β values calculated from the BB survival.

Statistical analysis was performed by two-way ANOVA testing in SPSS (version 26).

## 3. Results

**Figure 2:**
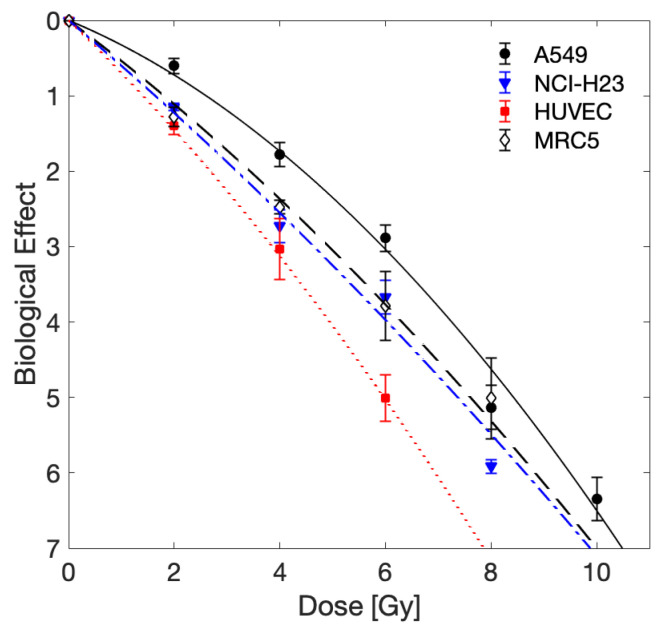
Experimentally measured biological effect of BB irradiation. Mean values and standard deviation of three independent experiments are shown. Data were fit by the LQ-model as indicated by lines (solid: A549, dashed: NCI-H23, dashed-dotted: MRC5, dotted: HUVEC); fit parameters obtained are shown in Table 1.

In order to compare the effectiveness of BB irradiation relative to MRT, we first established the sensitivity of the cell lines to standard BB radiation (Figure 2, Table 1). We observed that HUVECs (normal endothelial cells) were the most radiosensitive cells and A549 lung cancer cells were the most radioresistant. Statistical analysis revealed that at 2 Gy, survival of A549 cells was significantly higher (p<0.05) than any of the other three cell lines. No other significant differences in survival following BB irradiation were seen between any of the cell lines, at any of the given doses.

**Figure 3:**
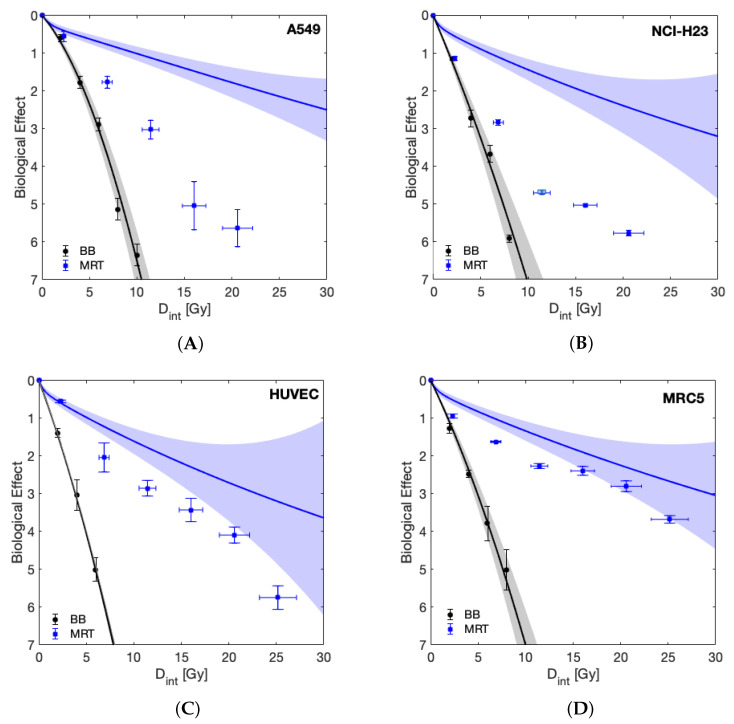
The experimentally measured biological effect of the investigated cell lines is plotted as a function of integral dose ($D_{int}$) for BB and MRT together with the surviving fraction predicted by the LQ-model using BB parameters (solid lines). Shaded areas represent uncertainty due to LQ-model parameter fit uncertainty and dosimetric uncertainty, as indicated in Figure 1. Data are shown for the tumour cell lines A549 (**A**) and NCI-H23 (**B**), and normal cell lines HUVEC (**C**) and MRC-5 (**D**).

Having established that the cell lines under study displayed comparable sensitivity to BB irradiation, we next evaluated MRT irradiation sensitivity, and predicted survival based upon the LQ-model with BB parameters (Equation (2)). Figure 3 shows the survival of the four cell lines following either BB or MRT, as well as the LQ-model-based predicted survival with relevant uncertainty bands. For all cell lines, BB irradiation was more effective than MRT when compared at equal integral dose levels. This behaviour was expected given the nonlinear dependence of cell survival on dose, implying that the mean of the surviving fractions of a dose spectrum deviates from the surviving fraction at the mean dose of that spectrum. A more meaningful comparison is drawn between the LQ-model prediction and the observed MRT cell survival. All four cell lines tolerated MRT less than predicted by the LQ-model with BB parameters. However, the clonogenic survival observed for the normal cell lines (MRC-5 and HUVEC) after MRT was closer to their predicted survival than the survival observed for the tumour cell lines, indicating enhanced tumour cell killing by MRT. In the case of the normal lung fibroblast cell line MRC-5, the observed survival fell within the uncertainties of the prediction at integral doses higher than 15 Gy.

**Figure 4:**
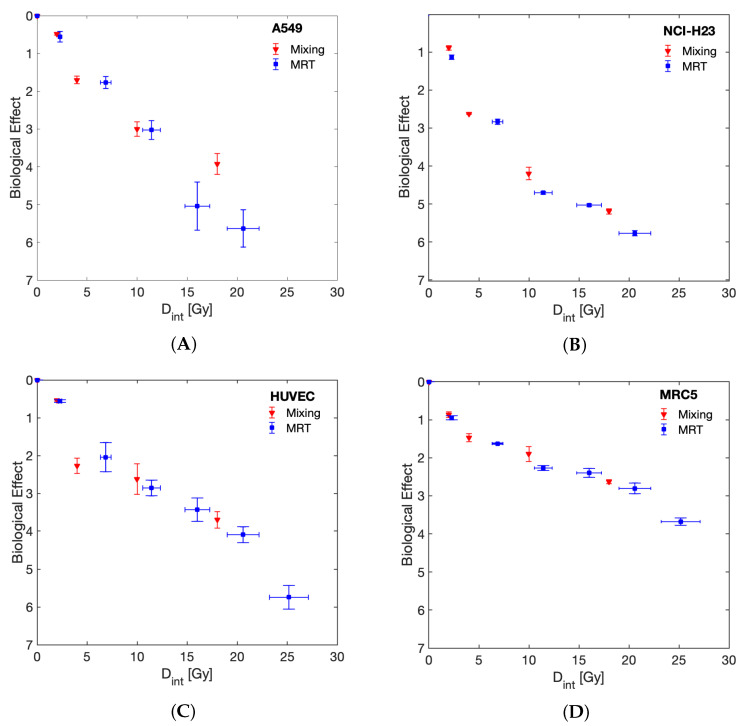
Comparing the biological effect of MRT and dose mixing experiments as a function of integral dose. Survival curves are shown for tumour (**A**) A549, (**B**) NCI-H23 and normal (**C**) HUVEC and (**D**) MRC-5 cell lines.

To assess the importance of the spatial distribution of dose gradients for MRT on the survival of normal and tumour cells, we performed dose mixing experiments, where cells were irradiated with BB irradiation at two dose levels and then the cells were mixed post irradiation with a PVDR of 22 and 80% of cells receiving the valley dose. Figure 4 demonstrates a response similar to that of MRT, as measured by clonogenic survival, that can be achieved in this way.

**Figure 5:**
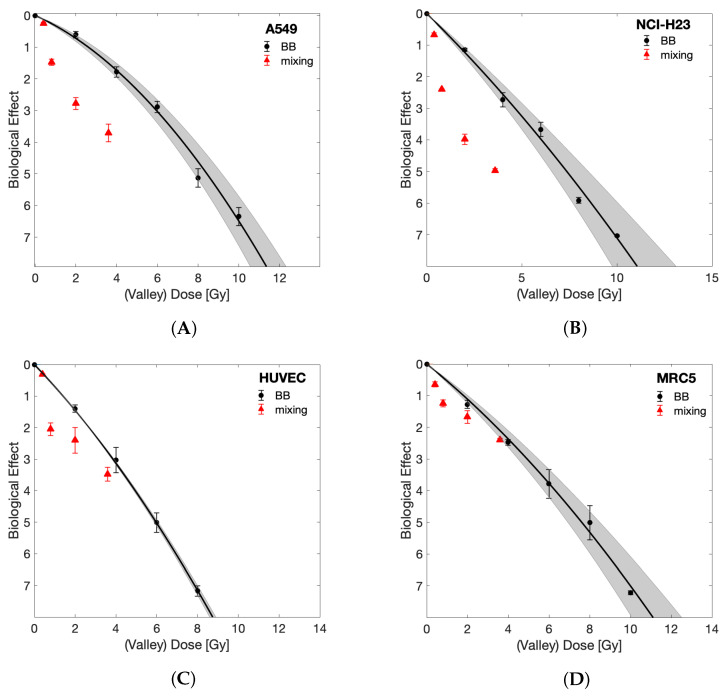
Comparing the biological effect of BB against the valley dose of dose mixing experiment. Dose mixing surviving fractions were divided by 0.8 to account for only 80% of cells receiving the valley dose. BB results are shown with the relevant LQ-model fit (shaded areas indicate 95% confidence intervals). Survival curves are shown for tumour (**A**) A549, (**B**) NCI-H23 and normal (**C**) HUVEC and (**D**) MRC-5 cell lines.

Figure 5 compares the clonogenic survival following BB irradiation and the valley dose (corrected to account for differences in seeding numbers) of the dose mixing experiments in order to evaluate differential response of normal and tumour cells in a direct comparison without model prediction. For all four cell lines, the survival of the cell population receiving the mixing valley dose was below that of the BB irradiated cells. Importantly, this difference in survival is much more pronounced for the tumour cells than the normal cells. Whereas in the two normal cell lines, the survival at the highest valley dose of 3.6 Gy falls within the margins of error of survival following BB irradiation (no significant difference), there was a significant difference for the A549 and NCI-H23 cells (p<0.01, unpaired *t*-test).

## 4. Discussion

In this study, we aimed to address three unmet research questions in the field of MRT: (i) is there a differential response of normal and tumour cell lines to MRT in vitro? (ii) Can the cellular response observed after synchrotron MRT be recapitulated using bench-top equipment and in the absence of FLASH effects, and (iii) how to best compare cell survival following BB irradiation to survival following exposure to inhomogeneous MRT dose distributions to obtain a biologically more meaningful representation compared to plotting as a function of either peak, valley or mean (i.e., integral) dose levels?

To date, there are few reports of a normal tissue sparing effect following spatially fractionated radiation at an in vitro level: whereas Ibahim et al. were unable to demonstrate normal tissue sparing [21], Peng et al. reported that the specific field patterns influenced the results and that 2.5 mm stripes but not 5 mm stripes resulted in decreased survival of tumour cell lines compared to homogeneous radiation [26]. Here, we demonstrated enhanced cell killing by MRT compared to BB irradiation (see Figure 3). This effect was significantly more pronounced in tumour cells compared to normal cells. As such, it indicates that the differential effect of MRT in vitro enhanced tumour cell killing rather than providing improved normal tissue sparing, as suggested by multiple studies in vivo [2,9,10,35,36,37]. It should be stressed that our results reflect clonogenic cell survival. Clonogenic assays are generally considered the gold-standard method for evaluation of radiosensitivity. For this assay, cells are trypsinised following irradiation (removing them from the spatial pattern) and plated at a relatively low density (≈5–1000 cells/cm${}^{2}$). In such a setting, cell–cell communication seems to be of importance after irradiation as a delayed event, as opposed to taking place during the radiation exposure itself. This would be in agreement with the accepted time frames of bystander signalling [38].

The spatial arrangement of the delivered dose was found here to be irrelevant for measured surviving fractions as the dose mixing experiments (Figure 4) demonstrate that the clonogenic survival following MRT can be replicated by separately irradiating cells with homogeneous peak or valley doses and mixing them post irradiation. This finding may be specific to the clonogenic assay, and it is possible that results would differ for assays where the cells remain in situ after irradiation or are plated at higher densities than those used for clonogenic assays.

To the best of our knowledge, we are the first to examine the effect of MRT in vitro using a bench-top X-ray source. The dose rates used here (<0.2 Gy/s) fall well below the range of dose rates previously attributed to FLASH effects [39]. Previous work employed synchrotron sources [21,26,40,41], and therefore, it has been impossible to distinguish if any differential effects of MRT and BB irradiation were due to the spatial fractionation or a result of FLASH effects. Here, we can discount any involvement of FLASH effects and attribute the differential response of normal and tumour cells wholly to the irradiation with high (peak) and low (valley) doses, either in the form of MRT or by post-mixing of separate BB irradiations. A similar conclusion was made by Smyth et al., who compared the relative toxicity of MRT and BB radiation at high and low dose rates [28]. They saw no evidence of normal tissue sparing following BB irradiation at dose rates of 37 to 41 Gy/s, which could be considered marginally below the range of dose rates typically associated with FLASH effects. Hence, we suggest that MRT, or rather the co-culture of peak and valley-dose irradiated cells, results in a differential response of tumour and normal cells at conventional dose rates. Importantly, this does not imply the absence of additional FLASH effects cumulative to a spatial fractionation effect in MRT experiments delivered using synchrotron facilities. Since conventional dose rates are not suitable for the delivery of MRT in vivo or within a clinical setting due to motion blurring of the dose profile, this analysis should be considered as a proof of principle only.

The work presented further contributes to the ongoing discussion on how to best compare the biological efficacy of MRT and BB as a function of “dose”. It is clear that due to the nonlinear dose relation of cell survival, neither the peak, valley nor integral dose alone can be used for this purpose. Instead, the full MRT dose spectrum needs to be accounted for. Here we provide two suitable methods for a direct BB and MRT comparison: 1. We included LQ-model predictions into the visualisation of our results (Figure 3) that account for the full dose-spectrum delivered to be able to compare MRT directly to the expected surviving fraction of the same dose spectrum in a BB setting. 2. Through dose mixing experiments, with known fractions of cells receiving only peak or valley doses, we were able to directly compare BB and spatially fractionated RT cell survival as a function of valley dose (with appropriate correction to account for only 80% of cells receiving this dose). The results obtained from these two approaches agreed and demonstrate an increased tumour cell killing in the presence of ablated cells. This implies that valley dose is not the only factor contributing to cell survival but that ablated cells negatively impact the survival of the population receiving the valley dose.

### Limitations of This Study

Our study is limited to the analysis of the differential response of tumour and normal cell lines to BB and MRT by clonogenic assays, and we did not further investigate the underlying mechanisms driving this differential response. We show that tumour cells were markedly more sensitive to mixed beam irradiations than normal cells, particularly when considering they showed equivalent sensitivity to BB irradiation. Importantly, we further demonstrate no significant differences in terms of clonogenic survival between MRT and co-culture experiments of ablated and surviving cell populations. Consequently, we expect the underlying mechanism responsible for the additional cell killing to be congruent with bystander signalling that was indicated by several previous analyses in co-culture experiments [42]. We suggest that the normal cell lines we used are less sensitive to bystander signalling than the tumour cell lines. It is well documented that not all cell lines are able to produce bystander signals [43,44] and are equally responsive to them. Specifically, both actively proliferating and transcriptionally active cells are more sensitive to bystander signals [45,46], which could cause a differential response to MRT, too. Future experimental analysis should address the mechanisms underlying the cellular response to MRT or mixed dose irradiation to confirm this hypothesis. We hope that our findings will motivate further discussion and experimental analysis in the community.

A second limitation of this study was the use of clonogenic assays. Despite frequent application for the characterisation of radiosensitivity, clonogenic assays represent a rather non-physiological cellular microenvironment of isolated cells plated on a rigid two dimensional (2D) substrate, limiting communication and inter-cellular contacts relative to the in vivo setting [47,48]. It would be important to investigate the presence of a differential response of normal and tumour cells to MRT in a more physiological setting, e.g., within 3D tumour spheroids [49,50]. Additionally, the use of in situ techniques, such as time-lapse microscopy, could allow an analysis of the impact of cell seeding density, and the spatial arrangement of ablated and surviving cells, on response.

Finally, the experimental arrangement used in this study posed some limitations on the delivery of BB and MRT. In order to maximise the field dose homogeneity for BB, and valley dose rate for MRT, different focal spot sizes and X-ray tube filaments were required. This implied differences in tube amperage to accommodate for the recommended specifications by the manufacturer. It should be noted that also the presence of the tungsten collimator may affect the photon spectrum in the case of MRT. Differential effects due to these variations in the photon energy spectra and dose rates between MRT and BB could not be accounted for. However, by achieving comparable cell surviving fractions, using MRT and dose mixing experiments may give an indication that the relevant differences were negligible in light of the biological uncertainty of clonogenic cell survival experiments. Furthermore, despite our best efforts, the dosimetric measurements were limited by the dynamic range and resolution of the used films. Hence, we show all of our results with the relevant dose uncertainties and accounted for these during the LQ-model predictions as well.

## 5. Conclusions

Using a bench-top X-ray source, we have demonstrated a differential in vitro response of lung cancer cell lines, endothelial cells and fibroblasts to microbeam irradiation at dose rates precluding the presence of FLASH effects. Specifically, we observed an increased tumour cell sensitivity to MRT, whereas normal cell survival following MRT was comparable to survival after BB irradiation. Cell survival after MRT was replicated by mixing populations of cells irradiated separately with high and low BB doses. These results provide a strong motivation for further investigations of the underlying mechanisms to confirm a potential role of bystander signalling.

## Figures and Tables

**Table 1:** LQ-model parameters α and β for homogeneous BB irradiation with 95% confidence bounds.

Cell Line	α $\left({\mathrm{Gy}}^{-1}\right)$	β $\left({\mathrm{Gy}}^{-2}\right)$
MRC5	0.52±0.06	0.018±0.007
NCI-H23	0.59±0.07	0.012±0.009
A549	0.29±0.05	0.036±0.006
HUVEC	0.67±0.01	0.028±0.002

## Data Availability

The data presented in this study are available in the Appendix A, Table A1, Table A2 and Table A3.

## Abbreviations

The following abbreviations are used in this manuscript:

DVH	Dose rate volume histogram
RT	Radiation therapy
MRT	Microbeam radiation therapy
BB	Broad beam
OAR	Organ at risk
PVDR	Peak-to-valley dose ratio
LQ model	Linear quadratic model

## Appendix A. Tabulated Cell Survival Data

**Table A1:** Broad beam cell survival data for the four cell lines used. Mean values and standard deviations of three independent repeat experiments are reported.

Dose [Gy]	HUVEC	MRC5	NCI-H23	A549
0	1 ± 0	1 ± 0	1 ± 0	1 ± 0
2	0.25 ± 0.03	0.28 ± 0.04	0.32 ± 0.02	0.55 ± 0.06
4	0.048 ± 0.019	0.084 ± 0.008	0.065 ± 0.014	0.17 ± 0.03
6	0.007 ± 0.002	0.023 ± 0.01	0.026 ± 0.006	0.056 ± 0.01
8	0.001 ± 0.0001	0.007 ± 0.004	0.003 ± 0.0002	0.006 ± 0.002
10		0.001 ± 0.00005	0.001 ± 0.00002	0.002 ± 0.001

**Table A2:** Microbeam cell survival data for the four cell lines used. Mean values and standard deviations of three independent repeat experiments are reported.

Mean Dose [Gy]	HUVEC	MRC5	NCI-H23	A549
0	1 ± 0	0.5 ± 0.25	1 ± 0	1 ± 0
2	0.57 ± 0.02	0.3 ± 0.14	0.32 ± 0.02	0.57 ± 0.08
6	0.13 ± 0.05	0.09 ± 0.02	0.059 ± 0.004	0.17 ± 0.03
10	0.06 ± 0.01	0.035 ± 0.011	0.009 ± 0.0004	0.049 ± 0.012
14	0.03 ± 0.01	0.021 ± 0.006	0.007 ± 0.0002	0.006 ± 0.004
18	0.017 ± 0.004	0.01 ± 0.003	0.003 ± 0.0002	0.004 ± 0.002
22	0.003 ± 0.001	0.002 ± 0.001		
26	0.001 ± 0.0006	0.001 ± 0.0001		

**Table A3:** Mixed broad beam cell survival data for the four cell lines used. Mean values and standard deviations of three independent repeat experiments are reported.

Mean Dose [Gy]	HUVEC	MRC5	NCI-H23	A549
0	1 ± 0	1 ± 0	1 ± 0	1 ± 0
2	0.59 ± 0.01	0.42 ± 0.03	0.41 ± 0.02	0.62 ± 0.02
4	0.1 ± 0.02	0.23 ± 0.024	0.073 ± 0.001	0.18 ± 0.02
10	0.072 ± 0.029	0.15 ± 0.029	0.015 ± 0.002	0.05 ± 0.009
18	0.025 ± 0.005	0.072 ± 0.004	0.006 ± 0.0004	0.02 ± 0.005
